# Comparison of the Upper Marginal Neurons of Cortical Layer 2 with Layer 2/3 Pyramidal Neurons in Mouse Temporal Cortex

**DOI:** 10.3389/fnana.2017.00115

**Published:** 2017-12-21

**Authors:** Huan Luo, Kayoko Hasegawa, Mingsheng Liu, Wen-Jie Song

**Affiliations:** ^1^Department of Sensory and Cognitive Physiology, Graduate School of Medical Sciences, Kumamoto University, Kumamoto, Japan; ^2^Program for Leading Graduate Schools HIGO Program, Kumamoto University, Kumamoto, Japan; ^3^Department of Neurology, Peking Union Medical College Hospital, Chinese Academy of Medical Sciences, Beijing, China

**Keywords:** neuronal morphology, regular spiking, intrinsic properties, synaptic depression, cortex anatomy

## Abstract

Layer 2/3 (L2/3) excitatory neurons in the neocortex make major contributions to corticocortical connections and therefore function to integrate information across cortical areas and hemispheres. Recent evidence suggests that excitatory neurons in L2/3 can have different properties. Sparse evidence from previous studies suggests that L2 neurons located at the border between L1 and L2 (referred to as L2 marginal neurons, L2MNs), have a morphology distinct from a typical pyramidal neuron. However, whether the membrane properties and input/output properties of L2MNs are different from those of typical pyramidal neurons in L2/3 is unknown. Here we addressed these questions in a slice preparation of mouse temporal cortex. We found that L2MNs were homogeneous in intrinsic membrane properties but appeared diverse in morphology. In agreement with previous studies, L2MNs either had oblique apical dendrites or had no obvious apical dendrites. The tufts of both apical and basal dendrites of these neurons invaded L1 extensively. All L2MNs showed a regular firing pattern with moderate adaptation. Compared with typical L2/3 pyramidal neurons that showed regular spiking (RS) activity (neurons), L2MNs showed a higher firing rate, larger sag ratio, and higher input resistance. No difference in the amplitude of excitatory and inhibitory postsynaptic potentials (EPSPs and IPSPs, respectively), evoked by stimulation of L1, was found between the two types of neurons, but the IPSPs in L2MNs had a slower time course than those in L2/3 RS cells. In paired recordings, unitary EPSPs showed no significant differences between synapses formed by L2MNs and those formed by L2/3 RS neurons. However, short-term synaptic depression (STSD) examined with a L2MN as the presynaptic neuron was greater when another L2MN was the postsynaptic neuron than when a L2/3 RS neuron was the postsynaptic neuron. The distinct morphological features of L2MNs found here have developmental implications, and the differences in electrophysiological properties between L2MNs and other L2/3 pyramidal neurons suggest that they play different functional roles in cortical networks.

## Introduction

The neocortex has six layers and contains distinct neuronal subtypes that enable the cortex to perform complex tasks (reviewed in Douglas and Martin, 2004). Each layer of the cortex has both excitatory and inhibitory neurons; overall, the majority of neurons are excitatory, and 15%–20% are inhibitory interneurons (Beaulieu, 1993). Excitatory neurons are primarily of pyramidal morphology and often exhibit regular spiking (RS) behavior in response to a constant current input, while inhibitory neurons are more diverse in morphology and electrophysiological properties (reviewed in Connors and Gutnick, 1990; DeFelipe et al., 2013).

In rodents, there is no clear architectonic boundary between layer 2 (L2) and L3 of the cortex, and these layers are therefore often referred as L2/3 (Peters et al., 1985; Lefort et al., 2009; Petersen and Crochet, 2013). L2/3 excitatory neurons in the neocortex make major contributions to corticocortical connections, including callosal connections, and therefore function to integrate information across cortical areas and hemispheres. Recent studies have shown that pyramidal neurons in L2/3 exhibit distinctive morphological (reviewed in Feldmeyer, 2012) and electrophysiological features in rodent neocortex (Lefort et al., 2009; Oviedo et al., 2010; Yamashita et al., 2013; Tyler et al., 2015). In sensory cortices, different L2/3 neurons have been shown to have different sound-responsiveness (Oviedo et al., 2010) or visual selectivity (Gur and Snodderly, 2008). Thus, L2/3 neurons are diverse in both structure and function. While the majority of cells in L2 are small pyramidal cells in the neocortex (Sholl, 1956; Winguth and Winer, 1986), stellate cells and fan cells have been reported in L2 of the entorhinal cortex, which is considered as the transition between three-layered allocortex and six layered neocortex (Canto et al., 2008; Tsuno et al., 2013; reviewed in Moser et al., 2010; Witter et al., 2017). In the neocortex, cells located at the border between L1 and L2 appear to have distinct morphology. Although these neurons have seldom been the subject of study, the few examples reported so far suggest that they have either no apical dendrite (Larkman and Mason, 1990) or only oblique apical dendrites (Peters and Kara, 1985; Cho et al., 2004; Staiger et al., 2015), unlike typical cortical pyramidal neurons, which have a single apical dendrite ascending towards the pia (Spruston, 2008). It is not known whether and how the electrophysiological properties of these neurons differ from other pyramidal neurons in L2/3. We refer to these neurons as L2 marginal neurons (L2MNs) and herein we studied their morphology, intrinsic membrane properties, and input/output properties, and compared L2MNs with other L2/3 pyramidal neurons in mouse temporal cortex. By combining multiple whole-cell patch-clamp recording and intracellular staining, we found that L2MNs are homogeneous in intrinsic membrane properties but diverse in morphology. We further found that L2MNs had intrinsic membrane properties and input/output features distinct from other L2/3 pyramidal neurons.

## Materials and Methods

### Slice Preparation

Postnatal day (P) 14–P21 C57BL/6J mice of either sex were used. All experimental procedures were approved by the Committee for Animal Experiments of Kumamoto University and followed the Guidelines for Use of Animals in Experiments of Kumamoto University. Mice were anesthetized with diethyl ether. After decapitation, the brains were removed quickly and placed in ice-cold artificial cerebrospinal fluid (ACSF: 126 mM NaCl, 10 mM Glucose, 26 mM NaHCO_3_, 2.5 mM KCl, 1.25 mM NaHPO_4_, 1 mM MgSO_4_, 2 mM CaCl_2_; pH 7.4, bubbled with 95% O_2_ and 5% CO_2_; 300 ± 5 mOsm/l). The brains were blocked for slicing by making two coronal cuts; one was made to remove the cerebellum and the other was made to remove the anterior pole of the brain (about 30% of the brain). The posterior surface of the block was glued onto the cutting stage of a vibratome (Linearslicer Pro 7; Dosaka EM, Kyoto, Japan). The block was quickly immersed in cold ACSF bubbled with 95% O_2_ and 5% CO_2_ and slices were cut at a thickness of 300–350 μm. Slices containing the rostral tip of the hippocampus were selected and maintained in an incubation chamber with a water bath at 34°C for 15 min and then at room temperature until recording.

### Electrophysiology

**Figure 1:**
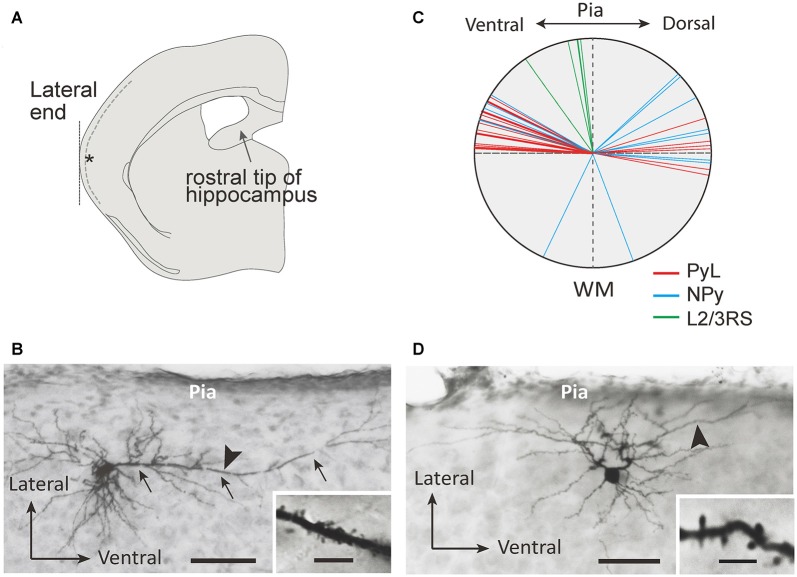
Morphological properties of L2 marginal neurons (L2MNs). **(A)** A schematic drawing of a coronal slice used for recording. Inclusion of the rostral end of the hippocampus was used as the criterion to select the slice; the target recording site is marked with the asterisk; the dotted line shows the border between L1 and L2. **(B,C)** Photomicrographs of a L2MN pyramidal-like (PyL; **B**) and a non-pyramidal (NPy) **(C)** neuron, intracellularly stained with biocytin. Arrows in **(B)** mark the “apical” dendrite. Scale bar = 100 μm. Insets in **(B,C)**: high magnification images of the dendritic segments marked by the arrowhead in **(B,C)**; the dendrites were covered with spines. Scale bar = 5 μm. **(D)** The apical dendrite orientation of PyL (red line; *n* = 22), NPy (blue line; *n* = 14) and L2/3RS (green line; *n* = 5) neurons.

Neurons were visualized under the microscope using infrared Nomarski optics (Stuart et al., 1993). At low magnification, the target recording site was located at the lateral end of the slice (see Figure 1A). Because of the sparsity of neurons in L1, L2MNs were easily identified as marginal cells of L2, facing L1. Whole cell recordings were obtained from single neurons or simultaneously from two or three neurons using Axopatch 200B (Molecular Devices, Sunnyvale, CA, USA). Because the purpose here was to compare L2MNs with L2/3 pyramidal neurons, a similar number of each type of neuron was recorded from slices of the same animal to suppress animal-dependent variance (Oswald and Reyes, 2008). Most recorded L2/3 pyramidal neurons were video-recorded for later analysis of soma position. The pipettes were pulled from glass tubes on a micropipette puller (Sutter Instruments, Novato, CA, USA), and had a resistance of 3–7 MΩ when filled with intracellular solution (128 mM gluconic acid potassium, 10 mM HEPES, 1 mM EGTA, 3.5 mM KCl, 0.1 mM CaCl_2_, 1 mM MgCl_2_, 2 mM Na_2_ATP, 0.2 mM LiGTP; pH 7.2–7.3; 280–290 mOsm/l). In current clamp mode, a family of step currents ranging from −50 pA to 490 pA (600 ms duration) in 60 pA increments were injected to examine cell intrinsic membrane properties; direct current was injected to keep the resting potentials to about −75 mV. In paired recordings, synaptic responses were studied in current clamp mode; the presynaptic cell was stimulated in current clamp mode by injecting suprathreshold current pulses (0.3–1.0 nA, 5 ms duration), and unitary postsynaptic responses were recorded in the postsynaptic cell. To study short-term synaptic dynamics, the presynaptic neuron was stimulated with a train of five suprathreshold current pulses (0.3–1.0 nA, 5 ms pulse width, 50 ms inter-pulse interval); the inter-trial interval was 5–10 s. All recordings were carried out at 34–35°C and the slices were continually perfused with ACSF oxygenated with 95% O_2_ and 5% CO_2_. In some paired recording experiments, the AMPA receptor antagonist 6-cyano-7-nitroquinoxaline-2, 3-dione (CNQX) (Tocris Bioscience, UK) was added to the external solution at a concentration of 10 μM.

For recording synaptic potentials evoked by stimulation of L1 (see below), the recording electrode was filled with a cesium-based solution containing 128 mM CH_3_O_3_SCs, 1 mM HEPES, 1 mM EGTA, 10 mM CsCl, 0.1 mM CaCl_2_, 1 mM MgCl_2_, 2 mM Na_2_ATP, 0.2 mM LiGTP, and 5 mM lidocaine *N*-ethyl bromide (QX-314; Sigma-Aldrich, USA), with pH adjusted to 7.2–7.3 using CsOH. This internal solution was used to make neurons electrotonically more compact.

### Electrical Stimulation of L1

While whole cell recordings were performed from L2MNs and L2/3 pyramidal neurons, electrical stimulation was applied to L1 at a distance below the pia one quarter of the thickness of L1, via a thin glass pipette filled with the extracellular solution. A single current pulse with a width of 80 μs was used for stimulation. The current level was gradually increased from zero to find the threshold to evoke a synaptic potential; a current level 40 μA above the threshold was used to evoke synaptic potentials in different cells for comparison.

### Histology

To visualize the recorded neurons, biocytin was always included in the intracellular solution at a concentration of 5 mg/ml. After recording, the slices were fixed with 4% formaldehyde in phosphate buffer (0.1 M; pH 7.4) for at least 24 h. The slices were further cut into serial sections of 70 μm thickness with a freezing microtome. Endogenous peroxidases were quenched with 1% H_2_O_2_ in Tris-buffered saline (TBS; 50 mM, pH 7.4) for 30 min. The sections were then rinsed in TBS three times (10 min each), treated with TBS containing 0.5% Triton X-100 (TBST), followed by the avidin-biotin-horseradish peroxidase reaction for 2 h, according to the manufacturer’s instruction (ABC-Elite; Vector Laboratories, Burlingame, CA, USA). Sections were washed in TBS three times and reacted with a mixture of diaminobenzidine tetrahydrochloride (0.04%) and nickel (II) ammonium sulfate hexahydrate (0.3%) in Tris-HCl (50 mM, pH 7.4) for 30 min. Last, 0.01% H_2_O_2_ was added for 3 min. After being rinsed with distilled water five times, the sections were mounted on gelatin-coated glass slides, counterstained with cresyl violet acetate for identification of cortical layers, dehydrated in a graded ethanol series, cleared in xylene, and cover-slipped with Entellan New (Merck, Darmstadt, Germany) for observation with a light microscope. The dendritic trees of some of the stained neurons were reconstructed from serial sections using Neurolucida (MBF Bioscience, Williston, VT, USA). Because parts of the dendrites were lost during slice preparation, the reconstruction reflects partial morphology of the cells. We did not wait long enough for biocytin to diffuse well into the axons as we were focused on dendrites.

### Data Analyses

AxoGraph (Molecular Devices, Sunnyvale, CA, USA) and Kaleidagraph (Albeck Software, Reading, PA, USA) were used for analyses of electrophysiological data. Data were presented as mean ± standard error of the mean (SEM), unless mentioned otherwise. Statistical difference between samples was tested using the Mann-Whitney *U* test. Significance was accepted when *p* < 0.05.

#### Intrinsic Membrane Properties

The resting membrane potential (V_rest_) was defined as the potential value upon membrane break in a whole cell recording (Kawaguchi, 1995; Joshi et al., 2015). The input resistance (R_in_) was estimated by injecting a small pulse current (10 pA). If the potential induced by the current rose to a stable plateau, fell to the baseline level with a time course similar to that of the rise phase, the potential was judged as lack of obvious active component and was used for calculation of Rin which was defined as the voltage change induced by the current at the plateau phase, divided by the current value. Hyperpolarization-activated component was tested by injecting a −50 pA pulse current, which induced a voltage sag. The sag ratio was calculated as (1 − (amplitude of steady-state hyperpolarization)/(peak amplitude of hyperpolarization)) × 100%.

#### Waveform Parameters

Waveform parameters for action potentials (APs) were measured for the first AP in a train of APs evoked by the minimum effective current, starting from −50 pA to 60 pA increments; the resting potential was controlled at −75 mV before current injection. Following Suter et al. (2013), we defined AP threshold as the voltage where ∆V/∆t equals 10% of its maximum value. AP peak amplitude was measured from the AP threshold to the peak. AP half-width was calculated as the time-span at the half-maximum amplitude of the AP. After-hyperpolarization potential (AHP) was the difference from AP threshold to the peak hyperpolarized potential, and the AHP peak time was the time interval from AP peak to the peak of hyperpolarized potential.

#### Synaptic Potentials

The amplitude of evoked excitatory postsynaptic potentials (EPSPs) or evoked inhibitory postsynaptic potentials (IPSPs) was measured as the peak potential from the baseline. To suppress the effect of noise, the baseline was calculated as the average of a 5 ms recording before stimulus, and the peak value was the average in a 1 ms window around the maximum point. Rise time was the time from 5% to 95% of peak amplitude, and half-width was the time from 50% upward to 50% downward of the peak response of EPSPs or IPSPs. Decay time was the time from the peak to half the peak value time during the decay phase. The slope of synaptic potentials was defined as the maximum slope of the potential in the first 1.5 ms window of the upward phase, measured using AxoGraph. To measure the amplitude of EPSPs in a train, exponential fitting to the preceding EPSP was extrapolated to the current EPSP and was used as the “baseline”.

#### Apical Dendrite Orientation

A circle with a 50 μm radius was drawn, with its center set at the center of the cell soma. The orientation from the center to the point where the circle crossed the apical dendrite was defined as the apical dendrite orientation. For cells without an obvious apical dendrite, we defined the thickest dendrite originating from the soma as the “apical” dendrite.

## Results

### Dendritic Morphology of L2MNs

We recorded from L2MNs at a specific location of the temporal cortex to sample a similar population of cells in different animals. As shown in Figure 1A, we selected the coronal slice containing the rostral tip of the hippocampus and recorded from L2MNs at the lateral end (Figure 1A, asterisk). Because of the low density of cells in L1, L2MNs could be easily identified by eye, under near-infrared microscopy. In addition to L2MNs, we also recorded from other L2/3 pyramidal cells for comparison; these cells could be identified under infrared microscopy before recording by their apical dendrites running towards the pia, and during recording by their RS activity; we refer to these cells as L2/3RS neurons.

**Figure 2:**
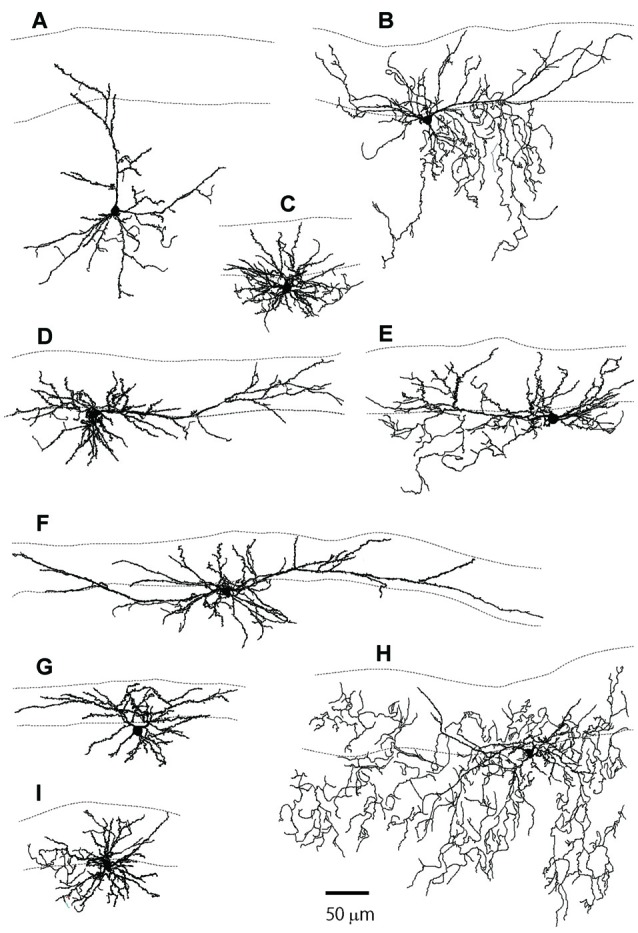
The reconstructed dendritic trees of L2MN PyL neurons, NPy neurons and a typical pyramidal neuron. **(A)** A typical pyramidal neuron with its apical dendrite running towards the pia. **(B,D–F,I)** Examples of PyL neurons with “apical” dendrites running parallel to the pia. **(C,G,H)** Examples of NPy neurons with dendrites extended in many directions, but with a thick primary dendrite that was defined as the apical dendrite for these cells. The two dotted lines in each figure show the pia surface and the border between L1 and L2, respectively.

Figures 1B,C show examples of L2MNs stained intracellularly with biocytin during recording. Partially reconstructed cell dendrites are shown in Figure 2 (Figures 1B,C, 2D,G show the same cells, respectively). The cell in Figure 1B had an “apical” dendrite (arrows), but the dendrite ran approximately along the border between L1 and L2; in other words, the dendrite ran parallel to the pia instead of towards it. More examples are illustrated in Figures 2B,E,F,I. The “basal dendrites” of such cells ramified in L2 as well as L1 (see Figures 2B,D–F,I), in contrast to a typical L2/3 pyramidal cell in which basal dendrites are virtually limited to L2/3 (Cho et al., 2004; Staiger et al., 2015; van Aerde and Feldmeyer, 2015; Figure 2A). Overall, these cells appeared like a typical pyramidal cell tilted 90° either towards the dorsal direction or the ventral direction. We called such cells L2MN pyramidal-like (L2MN PyL) cells. Other L2MNs, however, appeared to have dendrites extended in many directions, without a readily recognizable, long “apical” dendrite, such as the cell shown in Figures 1C, 1C, 2C,G,H. We refer to these cells as non-pyramidal (NPy) cells. PyL and NPy cells were identified by eye. NPy cells also had extensive dendritic arborizations in L1. The dendrites of both L2MN PyL and NPy cells were covered with spines, as shown in the insets in Figures 1B,C; this was confirmed in all cells successfully stained intracellularly (PyL: *n* = 22; NPy: *n* = 14). The most striking feature of L2MNs was the orientation of their “apical” dendrites; for NPy cells, we defined “apical” dendrite as the thickest primary dendrite. As shown in Figure 1D, the orientations of the “apical” dendrite (see “Materials and Methods” section for measurement of orientation) of all L2MNs were tilted more than 45° away from the pia (red lines and blue lines), in contrast to L2/3RS neurons, the orientation of which was mostly close to the pia (green lines; also see Figure 2A). For both L2MN PyL cells and NPy cells, some tilted towards the dorsal direction (PyL: 6/22; NPy: 8/14) and others tilted towards the ventral direction (PyL: 16/22; NPy: 6/14). We found no difference in orientation between PyL and NPy cells (PyL: 78.89 ± 2.47°, *n* = 22; NPy: 82.32 ± 9.31°, *n* = 14; *p* = 0.408; orientation counted from the pia towards ventral direction; all dorsal orientations flipped to ventral). The orientation of L2/3RS neurons (13.27 ± 5.36°; *n* = 5), however, was significantly different from both PyL (*p* < 0.0001) and NPy neurons (*p* = 0.0002).

### Intrinsic Membrane Properties of L2MNs

**Figure 3:**
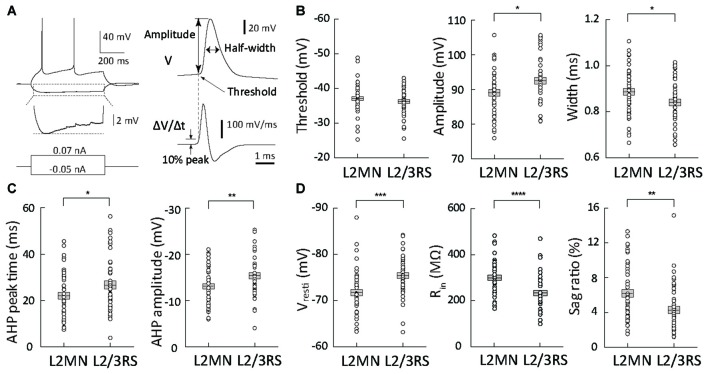
Comparison of action potential (AP) waveform parameters and passive membrane properties between L2MNs and L2/3RS neurons. **(A)** Measurement method of waveform parameters. Left: responses to injections of a −50 pA hyperpolarized current and a 70 pA depolarizing current that was the minimum current evoking an AP. The hyperpolarization response marked by the dotted box is further shown in an enlarged voltage scale to illustrate the sag potential. Right: the waveform of the first of the evoked APs was analyzed; the upper trace is the original recording (V) and the lower trace is the temporal differentiation of the upper trace (∆V/∆t). The time when the differentiated signal reached 10% peak value was used to measure the voltage value in the upper trace and the value was taken as AP threshold; the value was also used as “baseline” for measurement of AP amplitude. **(B)** AP waveform parameter values of L2MNs and L2/3RS neurons. Left: threshold. Middle: amplitude. Right: half-width. **(C)** After-hyperpolarization potential (AHP) parameter values of L2MNs and L2/3RS neurons. Left: AHP peak time. Right: AHP amplitude. **(D)** Passive properties of L2MNs and L2/3RS neurons. Left: Resting membrane potentials (V_rest_). Middle: Input resistances (R_in_). Right: Sag ratio. Error bar = standard error of the mean (SEM); *p < 0.05; **p < 0.01; ***p ≤ 0.001; ****p ≤ 0.0001.

The difference in morphology between L2MN PyL and NPy cells suggested possible differences in intrinsic membrane properties. To explore this possibility, we first compared V_rest_, R_in_ and sag potential to hyperpolarizing current injection (see “Materials and Methods” section for definition). As a result, we found no difference in V_rest_, R_in_, or sag between PyL and NPy cells (PyL: V_rest_ = −69.75 ± 0.99 mV, R_in_ = 256.6 ± 17.7 MΩ, sag = 0.73 ± 0.09 mV, sag ratio = 6.27 ± 0.70%, *n* = 20 for V_rest_, sag, and sag ratio, *n* = 19 for Rin; NPy, V_rest_ = −71.37 ± 0.91 mV, R_in_ = 249.0 ± 10.9 MΩ, sag = 0.75 ± 0.14 mV, sag ratio = 6.18 ± 1.03%, *n* = 12 for V_rest_, sag, and sag ratio, *n* = 10 for R_in_; *p* = 0.361 for V_rest_, *p* = 0.839 for R_in_, *p* = 0.687 for sag, and *p* = 0.566 for sag ratio; data not shown). Next, we compared AP waveforms of L2MN PyL and NPy neurons in terms of threshold, peak amplitude, half-width, AHP peak time, and AHP peak amplitude (see “Materials and Methods” section and Figure 3A for definitions). We found no significant difference in any of these parameters between L2MN PyL and NPy cells (threshold: PyL = −35.65 ± 0.73 mV, *n* = 20; NPy = −35.25 ± 1.41 mV, *n* = 12; *p* = 0.977; peak amplitude: PyL = 91.02 ± 1.58 mV, *n* = 20; NPy = 91.11 ± 1.12 mV, *n* = 12; *p* = 0.628; half-width: PyL = 0.89 ± 0.02 ms, *n* = 20; NPy = 0.83 ± 0.04 ms, *n* = 12; *p* = 0.114; AHP peak time: PyL = 25.8 ± 2.75 ms, *n* = 20; NPy = 26.61 ± 3.24 ms, *n* = 12; *p* = 0.888; AHP peak amplitude: PyL = −14.82 ± 0.90 mV, *n* = 20; NPy = −16.07 ± 0.69 ms, *n* = 12; *p* = 0.266). These results suggested that L2MN PyL and NPy cells are homogenous in intrinsic membrane properties. In the following experiments, we pooled data from the two groups into one L2MN group and compared these neurons with L2/3RS neurons.

### Comparison of Intrinsic Properties of L2MNs and L2/3RS Neurons

The locations of the soma of all video-recorded L2/3RS neurons (*n* = 34) were within 140 μm of the border between L1 and L2. A large proportion (71.6%) were within 100 μm of the border, a distance reported to reflect the border between L2 and L3 (Lefort et al., 2009; Petersen and Crochet, 2013). Thus, most L2/3RS neurons we recorded were in L2.

We first compared AP waveform parameters between L2MNs and L2/3RS neurons (Figure 3A). Compared with L2/3RS neurons, L2MNs had a similar AP threshold (L2MNs: −37.08 ± 0.58 mV, *n* = 51; L2/3RS: −36.28 ± 0.59 mV*, n* = 45; *p* = 0.335; Figure 3B, left), but a significantly smaller AP amplitude (L2MNs: 89.06 ± 0.94 mV, *n* = 51; L2/3RS: 92.50 ± 1.02 mV, *n* = 45; *p* = 0.015; Figure 3B, middle), and a significantly larger half-width (L2MNs: 0.89 ± 0.01 ms, *n* = 51; L2/3RS: 0.84 ± 0.01 ms, *n* = 45; *p* = 0.026; Figure 3B, right). AHP is an important factor affecting firing rate. Compared with L2/3RS neurons, L2MNs had a significantly shorter AHP peak time (L2MNs: 22.01 ± 1.41 ms, *n* = 51; L2/3RS: 26.63 ± 1.79 ms, *n* = 45; *p* = 0.043; Figure 3C, left) and a significantly smaller AHP amplitude (L2MNs: −13.09 ± 0.57 mV, *n* = 51; L2/3RS: −15.41 ± 0.68 mV, *n* = 45; *p* = 0.010; Figure 3C, right).

Compared with L2/3RS neurons, L2MNs had significantly less hyperpolarized V_rest_ values (L2MNs: −71.76 ± 0.67 mV, *n* = 51; L2/3RS: −75.46 ± 0.64 mV, *n* = 45; *p* = 0.0001; Figure 3D, left), significantly larger R_in_ values (L2MNs: 304.2 ± 11.2 MΩ, *n* = 43; L2/3RS: 237.9 ± 11.8 MΩ, *n* = 42; *p* < 0.0001; Figure 3D, middle), and a significantly larger sag potential in response to hyperpolarizing current injection (L2MNs: sag = 0.87 ± 0.07 mV, sag ratio = 6.21 ± 0.46%, *n* = 51; L2/3RS: sag = 0.46 ± 0.05 mV, sag ratio 4.26 ± 0.41%, *n* = 45; *p* < 0.0001 for sag and *p* = 0.002 for sag ratio, Figure 3D, right). Neither V_rest_, nor R_in_ were found to be correlated with animal age, for both L2MNs (*n* = 51, *p* = 0.070 for V_rest_; *n* = 43, *p* = 0.248 for R_in_) and L2/3RS neurons (*n* = 45, *p* = 0.283 for V_rest_; *n* = 42, *p* = 0.158 for R_in_).

**Figure 4:**
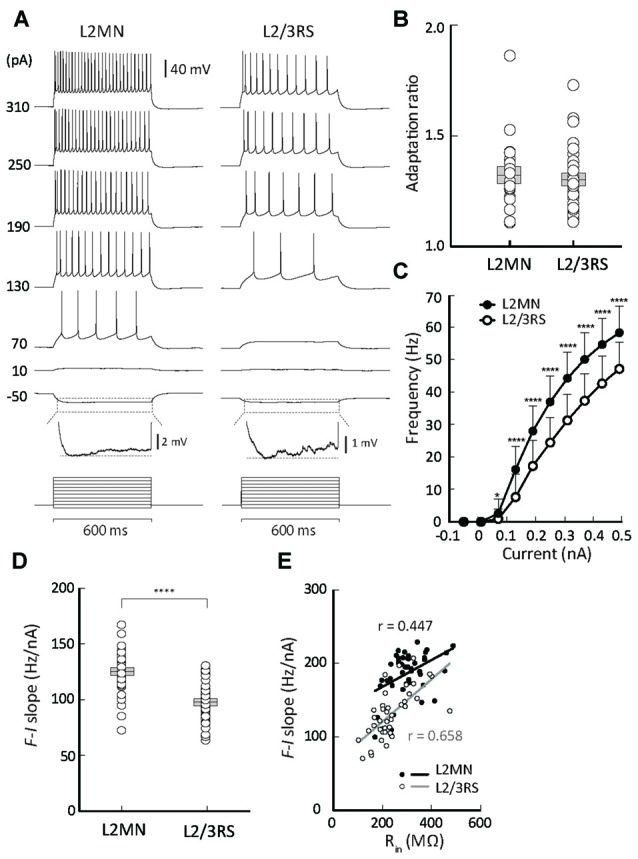
Comparison of firing properties between L2MNs and L2/3RS neurons. **(A)** Representative recordings from a L2MN and a L2/3RS neuron responding to a family of step currents, as labeled to the left. For both the L2MN and the L2/3RS neuron, the hyperpolarization response marked by the dotted box is further shown in an enlarged voltage scale to illustrate the sag potential. **(B)** Comparisons of adaptation ratio between L2MNs and L2/3RS neurons. **(C)** Comparisons of frequency-current relationships between L2MNs (black circles) and L2/3RS neurons (white circles). Error bar = SD; *p < 0.05; ****p ≤ 0.0001. **(D)** Comparison of the slopes of the frequency-current relationship between L2MNs and L2/3RS neurons. Error bar = SEM; *p* ≤ 0.0001. **(E)** Correlation between input resistance (R_in_) and the slopes of the frequency-current relationship (F-I slope) in L2MNs and L2/3RS neurons. A significant correlation was found for both types of neurons (*p* = 0.003 for L2MNs and *p* < 0.0001 for L2/3RS).

We next compared the firing properties of L2MNs and L2/3RS neurons in response to injection of a family of currents. Both L2MNs and L2/3RS neurons fired APs in a regular manner with moderate adaptation (Figure 4A), consistent with a previous study showing that excitatory neurons in L2/3 exhibit an adapting RS pattern (van Aerde and Feldmeyer, 2015). For quantitative description of cell firing, the inter-spike interval (ISI) was measured as the interval between the peaks of neighboring spikes. The instantaneous firing frequency was calculated as the reciprocal of ISI, and the average frequency was obtained from all ISIs for each level of injected current. To quantify adaptation, we injected a step current that evoked 10–12 APs, and calculated the adaptation ratio, according to the definition of Cho et al. (2004), as the ratio of ISI_9_ to ISI_3_. The adaptation ratio in L2MNs and L2/3RS neurons is shown in Figure 4B. No significant difference was found between the two cell groups (L2MNs: 1.32 ± 0.04, *n* = 19; L2/3RS: 1.31 ± 0.03, *n* = 29; *p* = 0.802; Figure 4B). According to the 1.67 criterion of the adaptation ratio (Cho et al., 2004; Staiger et al., 2015), both types of neurons belong to the slow-adapting type I subclass (Figure 4B).

With increasing amplitude of injected current, both L2MNs and L2/3RSs showed a monotonic increase in firing frequency (Figures 4A,C). At all levels of suprathreshold current, L2MNs showed a significantly higher average frequency of firing than L2/3RS neurons (Figure 4C; L2MNs, *n* = 51; L2/3RS, *n* = 45; *p* < 0.05 for 70 pA and *p* < 0.0001 for all currents >70 pA). Further, the slope of the frequency-current (F-I) curve, defined as the ratio of the frequency change in response to the change in current from 70 pA to 250 pA, was significantly larger in the L2MNs than in L2/3RS neurons (Figure 4D; L2MN: 124.90 ± 2.39 Hz/nA, *n* = 51; L2/3RS: 97.66 ± 2.61 Hz/nA, *n* = 45; *p* < 0.0001).

To explore the reason for the difference in the F-I relationship between the two types of neurons, we examined whether and how the intrinsic membrane properties were related to the slopes of the F-I curve (i.e., sensitivity of firing to current). We found that R_in_ values were positively related with the F-I curve slopes, in both L2MNs and L2/3RS neurons (L2MNs, *r* = 0.447, *n* = 43, *p* = 0.003; L2/3RS, *r* = 0.658, *n* = 42, *p* < 0.0001; Figure 4E).

### Synaptic Responses in L2MNs and L2/3RS Neurons to Electrical Stimulation of L1

**Figure 5:**
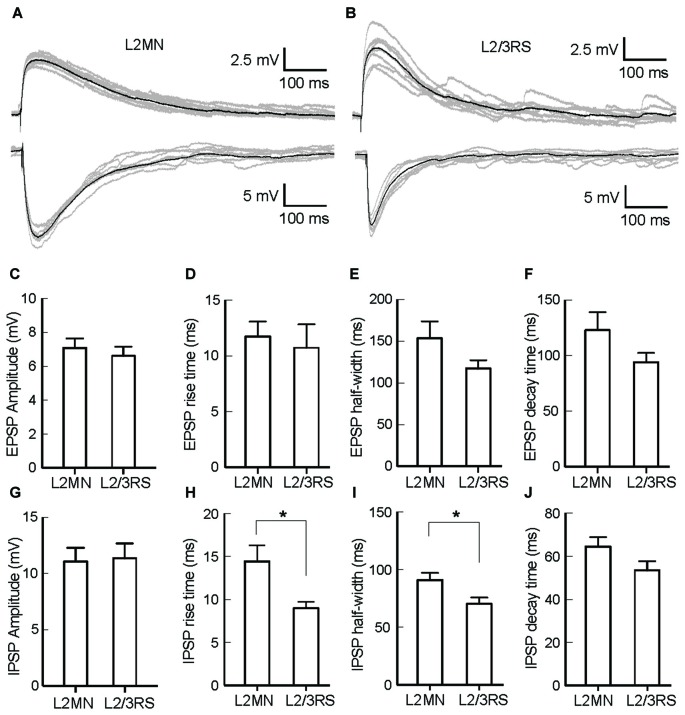
Comparison of synaptic responses evoked by stimulation of L1 in L2MNs and L2/3RS neurons. **(A,B)** Examples of EPSPs (Top) and IPSPs (Bottom) in a L2MN **(A)** and a L2/3RS neuron **(B)**. Gray traces are eight consecutive recordings, and the black traces are the average of the gray traces. **(C–F)** Comparisons of the amplitude, rise time, half-width and decay time, respectively, of EPSPs. Error bar = SEM. **(G–J)** Comparisons of the amplitude, rise time, half-width and decay time, respectively, of IPSPs. Error bar = SEM; *p < 0.05.

The ramification of “basal dendrites” of many L2MNs in L1 (see Figures 2B–I), in contrast to the sparsity of basal dendrites of L2/3RS neurons in L1 (see Figure 2A; Cho et al., 2004; Staiger et al., 2015) prompted us to speculate that L1 input has a different impact on L2MNs and L2/3RS neurons. To address this, we recorded synaptic potentials in L2MNs and L2/3RS neurons evoked by electrical stimulation of L1. We set the stimulation electrode in the upper half of L1 and recorded from both L2MNs and L2/3RS neurons along a line vertical to the cortical surface, passing through the position of the stimulation electrode. EPSPs were obtained by holding the membrane potential at the reversal potential of Cl^−^ (−60 mV), and IPSPs were isolated by holding the membrane potential at 0 mV, which is close to the reversal potential of cations (2.4 mV). As shown in Figures 5A,B, both EPSPs and IPSPs were evoked in L2MNs and L2/3RS neurons by a stimulus strength 40 μA above threshold. We found no difference between EPSPs in L2MNs and L2/3RS neurons in terms of amplitude (Figure 5C; *n* = 10 for L2MN, *n* = 10 for L2/3RS; *p* = 0.631), rise time (Figure 5D; *n* = 10 for L2MN, *n* = 10 for L2/3RS; *p* = 0.529), half-width (Figure 5E; *n* = 10 for L2MN, *n* = 10 for L2/3RS; *p* = 0.143), or decay time (Figure 5F; *n* = 10 for L2MN, *n* = 10 for L2/3RS; *p* = 0.123). For IPSPs, we found no difference between the two cell groups in terms of amplitude (Figure 5G; *n* = 7 for L2MN, *n* = 14 for L2/3RS; *p* = 0.799) or decay time (Figure 5J; *n* = 7 for L2MN, *n* = 14 for L2/3RS; *p* = 0.079). However, compared with L2/3RS neurons, L2MNs had a longer IPSP rise time (Figure 5H; *n* = 7 for L2MN, *n* = 14 for L2/3RS; *p* = 0.020) and a larger IPSP half-width (Figure 5I; *n* = 7 for L2MN, *n* = 14 for L2/3RS; *p* = 0.030).

### Properties of Synapses between L2MNs and L2/3RS Neurons

**Figure 6:**
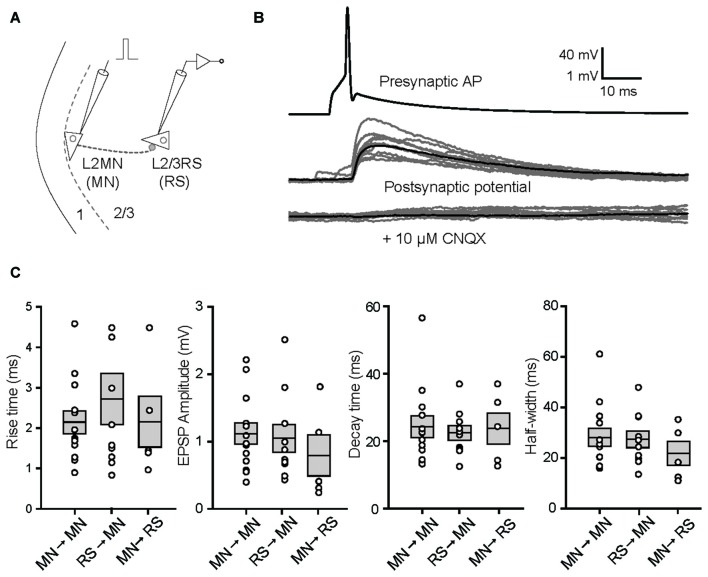
Properties of unitary synaptic responses obtained by multi-patch recording in pairs including a L2MN either as the presynaptic or as the postsynaptic neuron. **(A)** A schematic illustration of a paired recording; a short current pulse was injected into the presynaptic cell to evoke a single AP. **(B)** Examples of recordings from both the presynaptic neuron (top) and the postsynaptic neuron before (middle) and after application of 10 μM CNQX. In the middle and bottom panels, gray traces are from 10 consecutive recordings and the black trace in each of the panels is the average of 35 recordings including the gray traces. The pair is a L2MN→L2MN pair. **(C)** Comparisons of the rise time, amplitude, decay time and half-width of unitary EPSPs, among L2MN→L2MN, L2/3RS→L2MN and L2MN→L2/3RS pairs. Error bars are SEM. No significant differences were found.

Considering that most inhibitory interneurons have little number of dendritic spines other than the Martinotti cells (Kawaguchi et al., 2006), the spiny nature of L2MNs suggests that they are excitatory neurons. To verify this we did double patch recordings, with a L2MN as the presynaptic neuron and a L2/3RS neuron or another L2MN as the postsynaptic neuron (Figure 6A). Shown in Figure 6B is an example of a L2MN→L2MN pair (we use X→Y to denote a connection from X to Y). APs evoked in the presynaptic neuron (Figure 6B, top) elicited unitary postsynaptic potentials in the postsynaptic neuron, with no failure in this particular case (Figure 6B, middle). Application of CNQX (10 μM) drastically suppressed the potentials (Figure 6B, bottom), suggesting the glutamatergic nature of L2MNs. Similar observations were obtained in all four tested pairs.

We then compared the size and shape of unitary EPSPs evoked in L2MN-L2MN pairs and L2MN-L2/3RS pairs. In total we recorded from 273 L2MN-L2MN pairs, 21 of which had a connection; we also recorded from 378 L2MN-L2/3RS pairs, 16 of which had a L2/3RS→L2MN connection, and nine of the pairs had a L2MN→L2/3RS connection. We compared unitary EPSPs for all pairs where recording was stable and found no significant difference between L2MN→L2MN pairs and L2/3RS→L2MN pairs in terms of amplitude, rise time, decay time and half-width of unitary EPSPs (Figure 6C; amplitude: L2MN→L2MN = 1.12 ± 0.16 mV, *n* = 13; L2/3RS→L2MN = 1.05 ± 0.21 mV, *n* = 10, *p* = 0.648; rise time: L2MN→L2MN = 2.15 ± 0.28 ms, *n* = 13; L2/3RS→L2MN = 2.73 ± 0.63 ms, *n* = 10, *p* > 0.999; decay time: L2MN→L2MN = 24.36 ± 3.23 ms, *n* = 13; L2/3RS→L2MN = 22.51 ± 2.17 ms, *n* = 10, *p* > 0.999; half-width: L2MN→L2MN = 28.17 ± 3.53 ms, *n* = 13; L2/3RS→L2MN = 27.43 ± 3.27 ms, *n* = 10, *p* = 0.976). We also compared unitary EPSPs evoked in a L2MN and a L2/3RS neuron when a L2MN served as the presynaptic neuron. Again we found no significant difference between L2MN→L2MN pairs and L2MN→L2/3RS pairs in terms of amplitude, rise time, decay time, and half-width of unitary EPSPs (Figure 6C; amplitude: L2MN→L2MN = 1.12 ± 0.16 mV, *n* = 13; L2MN→L2/3RS = 0.81 ± 0.30 mV, *n* = 5, *p* = 0.246; rise time: L2MN→L2MN = 2.15 ± 0.28 ms, *n* = 13; L2MN→L2/3RS = 2.08 ± 0.61 ms, *n* = 5, *p* = 0.633; decay time: L2MN→L2MN = 24.36 ± 3.23 ms, *n* = 13; L2MN→L2/3RS = 23.87 ± 4.71 ms, *n* = 5, *p* = 0.503; half-width: L2MN→L2MN = 28.17 ± 3.53 ms, *n* = 13; L2MN→L2/3RS = 21.70 ± 4.72 ms, *n* = 5, *p* = 0.633).

**Figure 7:**
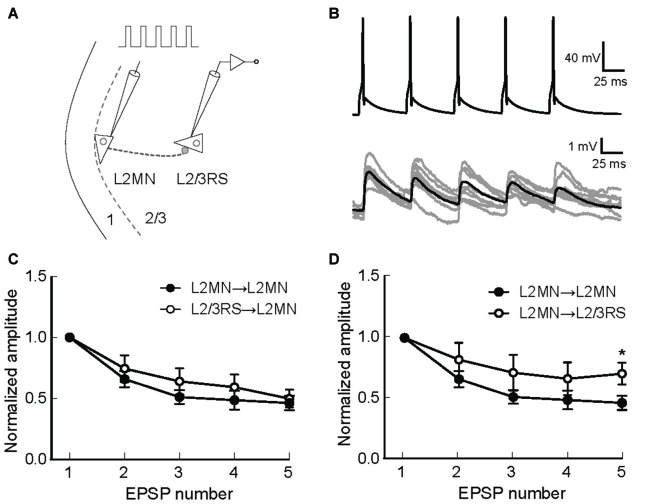
Short-term synaptic dynamics of synapses formed by L2MNs and synapses formed on L2MNs. **(A)** A schematic drawing of the stimulation and recording methods. Five current pulses were injected into the presynaptic neuron to evoke APs at approximately 20 Hz. **(B)** An example of recordings from a L2MN→L2MN pair. The upper panel shows APs evoked in the presynaptic neuron and lower panel shows EPSPs evoked by the presynaptic APs in the postsynaptic neuron. Gray traces are from eight consecutive recordings and the black trace is the average of 16 recordings including the eight gray traces. **(C)** Short-term dynamics of synapses formed onto L2MNs. EPSP amplitude was normalized to that of the first one. Depression was found for both L2MN→L2MN synapses and L2/3RS→L2MN synapses. No significant difference was found in the extent of depression. **(D)** Short-term dynamics of synapses formed by L2MNs. Stronger depression was found for the 5th EPSP when a L2MN neuron was the postsynaptic neuron, compared to the case where a L2/3RS neuron was the postsynaptic neuron. Error bar = SEM; *p < 0.05.

The above results suggest that L2MNs and L2/3RS neurons evoked similar synaptic potentials in each other when they fired a single AP. It is, however, more likely that these neurons fire trains of more than one APs during the execution of a cortical function. Thus, short-term synaptic dynamics would be an important property of these neurons. We first compared this property between synapses formed by L2MNs and synapses formed by L2/3RS neurons, both onto a L2MN. To this end, we evoked five APs at 20 Hz in a train in the presynaptic neuron by injecting short current pulses (Figures 7A,B) and simultaneously recorded the postsynaptic responses in a L2MN, using multi-patch recording. Both L2MN→L2MN synapses (*n* = 11) and L2/3RS→L2MN synapses (*n* = 7) exhibited synaptic depression and no significant difference was found between the two types of synapses (Figure 7C; *p* > 0.05 for all EPSPs). Next, due to the observation of target-dependent differences in short-term synaptic dynamics of neuronal interconnections in the cortex (Markram et al., 1998; Reyes, 2012; Joshi et al., 2015), we compared the short-term synaptic depression (STSD) between L2MN→L2MN and L2MN→L2/3RS synapses. As shown in Figure 7D, L2MN→L2MN synapses showed stronger depression than L2MN→L2/3RS synapses; the 5th EPSP (normalized to the 1st in Figure 7D) was significantly smaller in the L2MN→L2MN synapse than the L2MN→L2/3RS synapse (L2MN→L2MN: 0.46 ± 0.06, *n* = 11; L2MN→L2/3RS, 0.70 ± 0.09, *n* = 5; *p* = 0.038).

## Discussion

In this study, we found that, compared with a typical pyramidal cell, L2MNs had two distinct morphological features: the obliquity of “apical” dendrites and the ramification of “basal” dendrites in L1. Electrophysiologically, we found that L2MNs showed a regular firing pattern with moderate adaptation, with a higher firing rate, a more depolarized V_rest_, and a higher R_in_, when compared with L2/3RS neurons. IPSPs evoked in L2MNs by stimulation of L1 were found to have a slower time course but the same amplitude when compared with those evoked in L2/3RS neurons. We further found in multi-patch recordings that L2MN→L2MN synapses exhibited greater STSD than L2MN→L2/3RS synapses. Lastly, the spiny dendrites of L2MNs and the excitatory nature L2MN→L2MN/L2/3RS synapses found here strongly suggest that L2MNs are excitatory neurons, although the possibility that L2MNs include inhibitory interneurons cannot be completely excluded, in light of the existence of RS inhibitory neurons (Kawaguchi, 1995) and inhibitory neurons with spiny dendrites (Kawaguchi et al., 2006). Taken together, our findings suggest that L2MNs have distinct morphological and electrophysiological properties from other RS neurons in L2/3.

### Morphology of L2MNs

Our finding that L2MNs had oblique apical dendrites or no obvious apical dendrites is consistent with the sparse examples reported previously (Larkman and Mason, 1990; Cho et al., 2004; Staiger et al., 2015), and our study extended the number of examined neurons to 36. The morphology of NPy cells found here is similar to the stellate cells and fan cells found in L2 of the entorhinal cortex (Canto et al., 2008; Tsuno et al., 2013; reviewed in Moser et al., 2010; Witter et al., 2017), and the morphology of PyL cells found here is similar to the obliquely oriented pyramidal cells found in L2 of the medial entorhinal cortex (Klink and Alonso, 1997; Canto and Witter, 2012).

The distinct dendritic tree features of L2MNs compared with L2/3RS neurons have developmental implications, but the mechanism by which L2MNs develop distinct morphological features is unknown at this time. One possibility might be related to the substrate on which the apical dendrites grow (Jan and Jan, 2010); while the apical dendrites of other pyramidal cells in L2/3 grow in L2/3, the apical dendrites of L2MNs have to grow in L1 if they grow towards the pia. Our observation that “apical” dendrites of L2MNs ran along the border between L1 and L2 suggests that L2 might be more permissive for developing apical dendrites of pyramidal cells, including those of L2MNs. At the same time, our observations suggest that L1 might have repelling activity for the “apical” dendrites of L2MNs. These speculations need to be tested in future experiments. Given the apparent repelling activity of L1 for dendritic growth of pyramidal neurons, such an activity did not appear to affect the basal dendrites of L2MNs or the terminal portions of the “apical” dendrites of L2 neurons, because these dendrites or portion of dendrites were frequently found in L1 (see Figure 2).

Alternatively, the orientation of apical dendrites may be regulated by chemoattractants. It has been shown that Semaphorin 3A is required for the normal development of the orientation of apical dendrites towards the pia (Polleux et al., 2000). One may speculate that the distinct orientation of “apical” dendrites of L2MNs might be attributable to an inability of these dendrites to respond to Semaphorin 3A. If true, this will clearly set L2MNs apart from other pyramidal cells.

### Inputs from L1 onto L2MNs and L2/3RS Neurons

The differences in distribution of dendritic trees in L1 between L2MNs and L2/3RS neurons prompted us to test if synaptic responses evoked in these neurons by activation of L1 were different. We found that electrical stimulation of L1 evoked both EPSPs and IPSPs. While in general it is difficult to identify the exact element activated by the electrical stimulation (Ranck, 1975), the EPSPs are likely attributable, at least in part, to fibers from the thalamic matrix to L1 (Jones, 1998), and IPSPs are likely attributable to endogenous neurons in L1 (Hestrin and Armstrong, 1996). Although our results showed no difference in the size and shape of EPSPs, IPSPs were found to have a longer rise time and a longer half-width (see Figure 5). Namely, the time course of IPSPs was slower in L2MNs than in L2/3RS neurons. The reason for such a slower time course is not clear, but we speculate that the difference in the time course of IPSPs between L2MNs and L2/3RS neurons might be attributable to the differences in dendrites on which L1 inhibitory neurons impinge. Considering that the apical dendrites of L2 pyramidal cells have large tufts in L1 (reviewed in Feldmeyer, 2012), it is more likely for a L1 neuron to synapse on the apical dendrites of a L2/3RS neuron. In contrast, the extensive ramification of basal dendrites of L2MNs in L1 found here suggests a high probability for a L1 neuron to synapse on the “basal” dendrites of a L2MN. The slower time course of IPSPs in L2MNs might be attributable to a stronger filtering effect of basal dendrites than apical dendrites. A stronger filtering effect would also reduce the amplitude of unitary IPSPs. Thus, our observation of a similar amplitude of evoked IPSPs in L2MNs and in L2/3RS neurons might suggest a greater number of unitary IPSP inputs from L1 onto L2MNs than onto L2/3RS neurons. Such a speculation is consistent with the observation of extensive ramification of L2MN basal dendrites in L1 (see Figure 2). Other possible reasons for the slower time course of IPSPs in L2MNs than in L2/3RS neurons include that the types of L1 inhibitory neurons projecting to L2MNs and L2/3RS neurons are different; four types of inhibitory neurons in L1 have been identified (Hestrin and Armstrong, 1996; Wozny and Williams, 2011), and each type of them might induce different responses in L2MNs and L2/3RS neurons.

### Passive and Active Membrane Properties of L2MNs Compared with Those of L2/3RS Neurons

Electrophysiological properties of neurons are a key element for understanding neocortical network behavior (Contreras, 2004). Here we found that, compared with L2/3RS neurons, L2MNs had a less negative V_rest_, a larger R_in_, and a larger sag potential (see Figure 3). Despite the smaller amplitude and wider half-width of APs in L2MNs, they fired at higher frequencies than L2/3RS neurons (see Figures 3, 4). The higher firing rate of L2MNs may in part be attributable to their larger R_in_. A larger R_in_ produces a larger voltage in response to input of the same current, thereby producing a higher firing rate. The positive correlations between Rin and the slope of frequency-current curves found here for both L2MNs and L2/3RS neurons (Figure 4E) suggest that the larger slope values in L2MNs might be attributable in part to the larger R_in_ values in these neurons.

In addition to R_in_, the larger sag potential in L2MNs may also contribute in part to their higher firing rate. Although immunostaining of hyperpolarization-activated and cyclic-nucleotide-gated channel subunit 1 (HCN1) revealed no signal in rat L2/3 cortical neurons (Lörincz et al., 2002), we found both L2MNs and L2/3RS neurons exhibited a “sag” potential indicative of activation of the hyperpolarization-activated inward current (I_h_; Christophe et al., 2005; Sheets et al., 2011). I_h_ is expected to activate during the hyperpolarization phase to depolarize the membrane potential and thereby accelerate firing (Lüthi and McCormick, 1998; Robinson and Siegelbaum, 2003).

### Short-Term Synaptic Dynamics

In the present study, we found no difference in the amplitude and time course of unitary EPSPs elicited by a L2MN in another RS neuron and those elicited by a L2/3RS neuron in another RS cell. The STSD found here for synapses between L2MN and L2/3RS neurons is consistent with previous observations that excitatory synaptic connections between cortical pyramidal neurons usually show depression (reviewed in Reyes, 2012; Blackman et al., 2013). Further, we found that L2MN→L2MN synapses showed greater STSD than L2MN→L2/3RS synapses (see Figure 7D). It has been shown in the cortex that while short-term facilitation occurs in the pyramidal→interneuron synapse, STSD occurs in synapses formed by the same pyramidal neuron onto another pyramidal neuron (Markram et al., 1998). Such findings demonstrate target dependent, qualitative differences in synaptic transmission between synapses formed by the same presynaptic neuron. Our findings suggest pyramidal-cell-type-dependent, quantitative differences in synaptic depression between synapses formed by L2MNs. Our results are consistent with previous findings that excitatory synapses formed by pyramidal neurons in the cortex exhibit STSD to a different extent or no depression, depending on the subtype of postsynaptic pyramidal neurons (Atzori et al., 2001; Lee et al., 2014; Joshi et al., 2015), although the difference found here is more moderate than those reported previously.

Lines of evidence accumulated during the past decade suggest an emerging concept that the projectional identity of cortical pyramidal neurons is a primary factor determining membrane and synaptic properties (Morishima and Kawaguchi, 2006; Brown and Hestrin, 2009a, b; Anderson et al., 2010; Dembrow et al., 2010; Little and Carter, 2013; Shepherd, 2013; Yamashita et al., 2013). Whether the differences observed here between L2MNs and L2/3RS neurons are attributable to differences in projectional identity is unknown. A recent anatomical study showed that L2 neurons at the border between L1 and L2 (i.e., the L2MNs defined here) in most areas of the neocortex project to the temporal association area (Figures 5C,D in Zingg et al., 2014). Thus L2MNs might have projection targets different from those of L2/3RS neurons.

Cortical neurons have been classified into subtypes according to various criteria, such as electrophysiological properties (Connors et al., 1982; McCormick et al., 1985; Connors and Gutnick, 1990; van Aerde and Feldmeyer, 2015), morphology (Kriegstein and Dichter, 1983; Peters et al., 1985; Kawaguchi, 1995), projection targets (Morishima et al., 2011; Custo Greig et al., 2013; Yamashita et al., 2013; Harris and Shepherd, 2015), neural precursor lineages (Tyler et al., 2015) and molecular markers (Chan et al., 2001; Hevner et al., 2003; Inoue et al., 2004; Nieto et al., 2004). Despite the differences identified here between L2MNs and L2/3RS neurons, we hesitate to define L2MNs as a subtype until further lines of evidence are obtained, such as responsiveness to Semaphorin 3A and differences in projection targets. In any case, the morphological and electrophysiological features of L2MNs identified here must be taken into account when one builds a realistic model of the cortex.

### Functional Considerations

In addition to the developmental implications arising from the morphology of L2MNs, our results also have functional implications. The frequency-current relationship identified here suggests that L2MNs can fire more APs than L2/3RS neurons in response to the same excitatory input. The larger steepness of the frequency-current relationship of L2MNs suggests that these neurons are more sensitive to changes in input strength compared with L2/3RS neurons. The slower time course of IPSPs in L2MNs, compared with those in L2/3RS neurons, suggests that L2MNs are more likely to be suppressed tonically during sustained activity of L1 inhibitory neurons. Conversely, during sustained activity of L2MNs, postsynaptic L2/3RS neurons would be activated in a more sustained manner than other L2MNs, due to the higher extent of STSD in L2MN→L2MN connections (see Figure 7D). STSD/facilitation is important for a variety of types of neural processing, including adaptive processes (Abbott and Regehr, 2004; Oswald et al., 2006; Rotman et al., 2011; Reyes, 2012). At the network level, the consequence of the quantitative difference in STSD observed here between the L2MN→L2MN synapse and the L2MN→L2/3RS synapse remains to be explored.

## Author Contributions

HL and W-JS conceived of and designed the study, analyzed data, drafted the manuscript and figures. HL, KH and ML acquired and analyzed data. W-JS obtained funding.

## Conflict of Interest Statement

The authors declare that the research was conducted in the absence of any commercial or financial relationships that could be construed as a potential conflict of interest. The reviewer MM and handling Editor declared their shared affiliation.
